# Identification of Acoustic Emission Spectrograms from Limestone Fracturing Based on a Novel Deep Learning Model

**DOI:** 10.3390/s26134157

**Published:** 2026-07-01

**Authors:** Yan Zhang, Daojing Guo, Yulong Ye, Lantao Huang, Cong Fan, Jiancheng Huang, Mingdong Wei

**Affiliations:** 1 Guangxi Key Laboratory of Geomechanics and Geotechnical Engineering, Guilin University of Technology, Guilin 541004, China; zhangyan@glut.edu.cn (Y.Z.); 1020230771@glut.com (Y.Y.); 2120230867@glut.com (L.H.); 2120230906@glut.edu.cn (C.F.); 2 Guangxi Key Laboratory of Green Building Materials and Construction Industrialization, Guilin University of Technology, Guilin 541004, China; 3 State Key Laboratory of Geohazard Prevention and Geoenvironment Protection, Chengdu University of Technology, Chengdu 610059, China; gdj@stu.cdut.edu.cn; 4 State Key Laboratory of Hydraulics and Mountain River Engineering, College of Water Resources and Hydropower, Sichuan University, Chengdu 610065, China; huangjiancheng@stu.scu.edu.cn

**Keywords:** deep learning, PCA-VGG16 model, acoustic emission, image recognition, acid-alkali pretreatment

## Abstract

The progressive development of microscopic fractures within rock masses is a primary mechanism of macroscopic failure, threatening the structural integrity of rock engineering systems. In this paper, a novel deep learning model, Principal Component Analysis (PCA)-Visual Geometry Group 16 (VGG16), is developed to accurately identify spectrogram features associated with limestone fractures. In this architecture, a PCA-based convolution encoder is seamlessly integrated as a foundational preprocessing layer before feedforwarding into the deep neural network to execute linear feature purification. The model is first validated on standard image datasets comprising handwritten digits and facial images to evaluate classification performance. Subsequently, acoustic emission signals are acquired during triaxial compression tests on limestone specimens pretreated with cyclic acid–alkali exposure. The PCA-VGG16 framework is then employed to classify the corresponding acoustic spectrograms, and its performance is quantitatively compared with a conventional convolutional neural network (CNN) and the standard VGG16 model. The results indicate that the PCA-VGG16 model achieves classification accuracies that are 19.19% and 10.77% higher than the conventional CNN and standard VGG16 models, respectively. In terms of computational efficiency, the training time is reduced by 35.00% and 23.53% compared to CNN and VGG16. The superior classification performance of the proposed PCA-VGG16 model enables accurate identification of internal microscopic fracture characteristics in limestone. Furthermore, the integration of acoustic emission signals with deep learning models offers an effective approach for quantifying internal fracture levels and predicting the progressive failure of rocks.

## 1. Introduction

The progressive initiation, propagation, and coalescence of microcracks are widely recognized as the fundamental mechanisms governing macroscopic rock failure, posing a direct threat to the long-term structural integrity of underground openings, tunnels, and deep excavations [1,2]. In many geological environments, the presence of water—particularly when accompanied by cyclic acid-base alterations—accelerates microcrack development, induces coupled chemical and physical deterioration, reduces the mechanical strength of rock, and may ultimately lead to catastrophic failure. Elucidating microcrack evolution is therefore essential for predicting macroscopic instability and preventing engineering disasters [3,4,5]. However, direct observation and quantitative characterization of these microscale cracks remain extremely challenging owing to their extremely small scale and highly heterogeneous distribution [6,7]. Moreover, the relationship between internal fracture progression and ultimate macroscopic failure modes under complex environmental conditions is still poorly understood, highlighting the urgent need for effective monitoring and identification methodologies.

Acoustic emission (AE) technology offers a promising non-destructive testing approach for capturing, in real time, the elastic waves released during crack initiation and propagation, thus serving as a powerful proxy for monitoring internal damage evolution in rock [8,9,10]. Through analysis of AE parameters such as counts, energy, amplitude, and frequency characteristics, researchers have attempted to correlate AE signal features with different fracture modes and damage stages [11,12]. For instance, tensile and shear cracks have been shown to generate distinctly different AE spectral signatures, enabling preliminary discrimination of failure mechanisms [13,14]. Concurrently, several studies have investigated the strength variations of rock subjected to cyclic wet–dry or acid-base pretreatment [15,16]. Despite these advancements, traditional AE analysis methods still mainly rely on manually selected parameters (or statistical indicators), which have problems such as limited sample size and subjective threshold selection, making it difficult to systematically process large amounts of continuous waveform data [17,18,19]. These limitations result in low classification accuracy and inefficient identification of rock failure stages, a problem that becomes particularly pronounced when rapid, automated processing of large quantities of AE spectrograms is required [20,21]. Consequently, there is an urgent need to develop more intelligent and robust classification models capable of learning discriminative features directly from AE spectrogram images.

Recent advances in deep learning have revolutionized image and spectral pattern recognition, with convolutional neural network (CNN) achieving state-of-the-art performance in numerous classification tasks [22,23]. This success has spurred the application of CNN-based models in geotechnical engineering [24]. In the field of AE monitoring, some researchers have transformed raw AE waveforms into spectrogram images via time-frequency analysis and subsequently employed CNN architectures to classify these images according to damage stages or fracture types [25]. For example, Zhang et al. [26] proposed a method that integrates AE signals with an information-fusion CNN for the semi-quantitative identification of pore characteristics in sandstone from the Yungang Grottoes. Song et al. [27] converted AE waveforms of rock fracturing under various loading conditions into time-frequency images and employed five CNN models to classify the AE signals, achieving high recognition accuracy. Among the available CNN architectures, VGG16 (Visual Geometry Group) stands out owing to its uniform 3 × 3 convolutional kernels and deep architecture, which enable the extraction of rich hierarchical features and deliver excellent performance in generic image recognition tasks [28,29,30]. The application of VGG16 has been extended to structural health monitoring and fault diagnosis, showing promising results in spectrogram pattern classification. For instance, Sun et al. [31] regarded rock fracturing AE signals as a type of speech signal and constructed six intelligent real-time recognition models based on VGG16 and AlexNet, providing a new approach for real-time intelligent identification of critical AE signals during rock failure. Ren et al. [32] used AE data from Brazilian splitting and direct shear tests on red sandstone, employed three CNN networks including VGG19 to classify waveform images and spectrograms for distinguishing tensile and shear signals, and found that CNNs significantly outperformed traditional decision tree methods, recommending the combination of VGG19 with waveform images as an effective means to discriminate tensile-shear fracture AE signals. However, directly applying VGG16 to the classification of rock AE spectrograms presents significant challenges. First, the high dimensionality of input spectrograms leads to excessive computational cost and long training times, limiting the model’s suitability for practical large-scale monitoring tasks. Second, redundant or noisy frequency components present in AE spectrograms can easily cause overfitting and degrade generalization capability. Furthermore, the fully connected layers of VGG16 contain an enormous number of parameters that require meticulous tuning, further increasing training complexity. As a result, when dealing with the inherently complex and variable AE signals of rock subjected to different chemical environments, the direct use of VGG16 often yields suboptimal accuracy and poor time efficiency. Enhancing the VGG16 architecture to achieve high-accuracy classification while reducing the computational burden is therefore an urgent research need.

To address the above challenges, this study proposes a novel cascaded learning framework (PCA-VGG16) that structurally integrates an upstream linear feature purification operator with a downstream topologically optimized deep network. In this architecture, the PCA-based component is presented as a PCA-based convolution encoder that serves as a foundational preprocessing layer of the entire framework before feedforwarding into VGG16. Rather than acting as an isolated preprocessing routine, this upstream convolution encoder is strictly constrained by a cumulative variance contribution rate (≥95%) to filter out global linear background noise, equipment baseline drifts, and mechanical friction correlations from the acoustic emission (AE) spectrograms. This streamlined, highly purified representation is subsequently mapped into a specialized VGG16 architecture for deep localized feature extraction and classification, thereby establishing an optimal Pareto trade-off between top-tier identification accuracy and ultra-low training latency by eliminating structural capacity surplus and redundant computations.

Limestone was selected as the study object because this rock type is widely distributed in underground engineering areas such as tunnels, mines, hydraulic complexes, and urban deep excavations. Its mineral composition is dominated by calcite, which exhibits extremely high chemical reactivity with acidic aqueous solutions. Under complex hydrochemical environments—such as acid rain, acidic groundwater, or chemical grouting leakage—cyclic acid-base attack and wet–dry alternation significantly accelerate the dissolution of carbonate minerals, microstructural loosening, and mechanical strength degradation, thereby seriously threatening the long-term reliability of engineering structures. Systematically investigating the fracture evolution characteristics and AE response of limestone subjected to acid-base and wet–dry cycles is therefore of clear engineering value and scientific significance. Subsequently, triaxial compression tests were conducted on limestone specimens subjected to cyclic acid-base exposure and wet–dry cycles, during which AE signals were continuously recorded. The captured AE waveforms were converted into 2D spectrogram images corresponding to different failure stages, and the proposed PCA-VGG16 model was used to classify these spectrograms. To establish the methodology’s engineering proficiency and methodological originality, the encoded representation and overall classification performance were rigorously benchmarked against both traditional networks (CNN and standard VGG16) and advanced alternative frameworks, including deep residual topologies (ResNet18), non-linear unsupervised compression schemes (Autoencoder-VGG16), and the dedicated PCA-FC ablation baseline.

## 2. PCA-VGG16 Acoustic Spectrum Recognition Model

### 2.1. Model Principle

**Figure 1:**
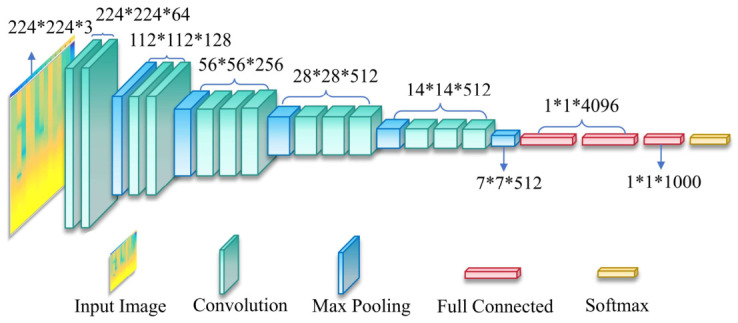
A schematic diagram of the VGG16 model.

The VGG16 model is an optimized version of the traditional CNN, established on the deep learning framework TensorFlow [33,34]. It comprises 13 convolutional layers, 3 fully connected layers, 5 pooling layers, and 1 regression layer (Softmax), as illustrated in Figure 1. However, due to the numerous convolutional and fully connected layers in VGG16, processing RGB three-channel images with this model results in extended training times and challenging parameter adjustments. To reduce the model’s time consuming operations and enhance classification accuracy, dimensionality reduction is employed during the VGG16 model classification [35,36]. Principal Component Analysis (PCA) is a non-parametric technique used for this purpose. The PCA can linearly transform a multivariate dataset with correlated components into an uncorrelated component dataset. The objective of PCA is to minimize the loss of feature information while retaining a significant portion of the original variables’ information. The PCA finds extensive applications in various fields, such as data compression and image enhancement [37,38].

**Figure 2:**
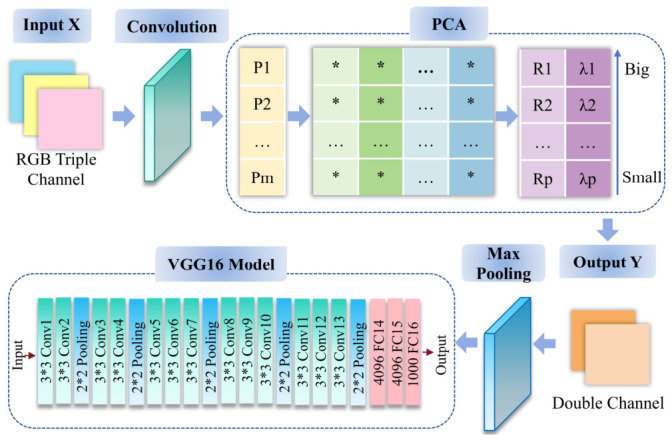
A schematic diagram of the proposed VGG16 model.

The PCA-VGG16 model aims to reduce the dimensions of the RGB three-channel image in the VGG16 model’s convolutional and pooling layers while preserving image features to the maximum extent. The process is illustrated in Figure 2. By employing PCA for dimensionality reduction of the image data, the training data becomes significantly simplified. This simplification reduces the size of the output parameters of the final fully connected layer in the model, leading to reduced parameter adjustment complexity and improved model efficiency. In this study, extensive parameter tuning is performed to identify the optimal model parameters, resulting in the highest classification accuracy. A comparison is made between the classification accuracy of the original VGG16 model (without dimensionality reduction) and the proposed PCA-VGG16 framework—which integrates PCA for feature purification and dimensionality reduction—to assess the effectiveness of PCA in enhancing the performance and computational efficiency of the VGG16 model.

It is worth noting that while PCA is a linear dimensionality reduction method, it may have limitations in capturing high-order nonlinear manifold features compared to nonlinear techniques such as Autoencoders or t-SNE. However, PCA offers distinct advantages in terms of extremely low computational complexity, independence from hyperparameter tuning, and clear physical interpretability. Given the stringent real-time requirements for geological disaster warning systems, PCA was selected in this study to achieve an optimal trade-off between feature representation capability and computational workload. Future research will explore the integration of nonlinear dimensionality reduction techniques with deep learning architectures to continuously optimize model performance.

In terms of feature preservation, this study maintained a high cumulative variance contribution rate (over 95%) during PCA implementation. This strategy ensures that while redundant high-frequency noise and hardware interference are filtered out—effectively functioning as a ‘feature purification’ process—the core energy-carrying structural textures essential for identifying rock fracture stages are rigorously preserved. Furthermore, while PCA is a linear transformation, the deep convolutional architecture of the subsequent VGG16 model is highly capable of learning hierarchical spatial features from the retained principal components. This deep learning capability effectively compensates for the potential loss of minor nonlinear details, thereby ensuring the robustness and high precision of the classification performance.

### 2.2. Model Development

The PCA-VGG16 model is an integrated framework synthesized through the collaborative coupling of a PCA-based convolution encoder and a topologically optimized Visual Geometry Group 16 (VGG16) deep neural network. The systemic architectural construction of this model progresses through the following sequential phases.

First, the PCA-based convolution encoder is seamlessly integrated into the network as the foundational preprocessing layer of the overall architecture before feedforwarding into VGG16, serving as the core operator for linear feature purification and structural encoding. Transcending the scope of conventional data preprocessing, this encoder layer executes an eigen-decomposition on the empirical covariance matrix of the acoustic emission (AE) signals, thereby mapping the high-dimensional manifolds of the raw AE spectrograms into a lower-dimensional orthogonal feature space. To rigorously suppress the redundant high-frequency noises induced by the hardware acquisition environment and mechanical friction of the loading system, while concurrently maintaining the integrity of the primary energy-carrying structural textures essential for characterizing rock fracture evolution, a stringent cumulative variance contribution rate threshold (≥95%) is established to dynamically filter the principal components. By functioning as an upstream linear encoder, this methodology substantially curtails the dimensionality of the input vectors, thereby alleviating the computational overhead and spatial complexity for the subsequent deep convolutional operations.

Subsequently, the purified low-dimensional principal component vectors encoded by this preprocessing layer are channeled into a modified VGG16 architecture characterized by localized topological streamlining and parametric de-redundancy. Given that the standard VGG16 network was originally engineered for the ImageNet dataset involving 1000 densely categorized classes, its terminal fully-connected segment exhibits severe parametric redundancy, traditionally comprising two massive dense layers with 4096 hidden units each. Direct transfer of such an unpruned configuration to our small-sample rock AE multi-stage classification task inevitably triggers the curse of dimensionality and catastrophic structural overfitting. To rectify this, a profound model-level optimization is applied to this computationally intensive block: the original dual 4096-node sequences are reconfigured into a cascading, lightweight dense architecture with 512 and 128 units, respectively, followed by a customized Softmax classification layer tailored explicitly to the rock failure stages. This architectural refinement shrinks the trainable parameter scale of the classifier by over 85%, significantly minimizing spatial complexity and safeguarding the network against generalization degradation induced by structural capacity surplus.

To establish robust optimization constraints under the small-sample regime inherent to empirical rock non-linear failure datasets, a multidimensional synergistic regularization and adaptive generalization control mechanism is embedded within the restructured classifier. Specifically, a Dropout mechanism with a predefined dropout rate of *p* = 0.5 is interleaved between the sparse dense layers to disrupt inter-neuronal co-adaptation. Concurrently, an L2 weight regularization penalty (with a weight decay coefficient optimized at 0.001) is integrated into the formulation of the empirical loss function, enforcing a bounded constraint on the parameter magnitudes to suppress weight explosion during backpropagation. For the optimization trajectory, the Adam algorithm is utilized alongside a categorical cross-entropy objective function, tightly coupled with an adaptive learning rate scheduler initialized at 0.001.

**Figure 3:**
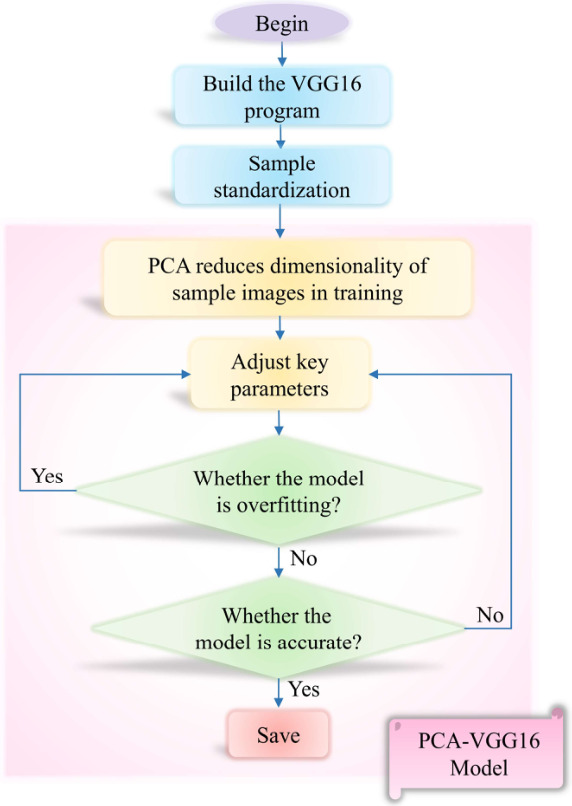
A flowchart of the main implementation procedures of the proposed PCA-VGG16 model.

To quantitatively monitor the network evolution status and optimize hyperparameter configurations, an adaptive closed-loop control mechanism is implemented within the execution flow, as illustrated in Figure 3. First, regarding key parameter tuning, this study co-optimizes the maximum number of training epochs and the batch size, wherein the batch size is adaptively calibrated between 16 and 128 based on the data scale and physical sample complexity. Secondly, the system adopts the dual-track accuracy gap as the quantitative criterion for evaluating whether the model is overfitting: the training and test accuracies are extracted synchronously at the end of each epoch; if the generalization gap between them widens significantly (i.e., the training accuracy approaches 100% while the independent test accuracy stagnates or degrades), the model is diagnosed as entering an overfitted state, which immediately triggers the active termination path shown in Figure 3. Concurrently, the convergence trajectory of the test loss is monitored in real time to execute the early stopping strategy, where the termination patience is strictly restricted to 5 epochs; if the test loss fails to decrease monotonically for 5 consecutive periods, training is actively terminated, and the restore best weights mechanism automatically discards the overfitted terminal parameters, restoring and preserving the global optimal weights achieved at the absolute nadir of the test loss curve. Finally, the system leverages the independent test accuracy as the definitive scale for final convergence evaluation. The finalized network is validated as accurate and generalization-reliable and subsequently exported only when its test accuracy stably surpasses a predefined high-recognition engineering threshold (≥90%) upon early stopping activation, hereby achieving the closed-loop evaluation cycle of Figure 3 and ensuring that the network efficiently converges toward the global optimum across the complex, non-linear rock fracture manifolds.

### 2.3. Accuracy Evaluation Index

In verifying neural network models, image classification is commonly used to assess the quality of classification and recognition. The effectiveness of model classification and recognition is evaluated by analyzing the accuracy of image classification and recognition. In this study, two indicators, namely classification accuracy and loss rate, are employed to assess the performance of various rock classification models. Classification accuracy refers to the ratio of correctly classified samples to the total number of training or test samples within the training set or test set, respectively. A higher classification accuracy indicates a better classification effect of the model. The specific formula for calculating the Classification Accuracy (*CA*) is as follows:

(1) $$CA=\frac{AS}{TS}\times 100\%$$

where *AS* represents the number of accurately classified samples (Accuracy Samples) in the training set (or test set), and *TS* represents the total number of samples (Total Samples) in the training set (or test set).

The loss rate (Loss Rate, LR) refers to the convergence value of the loss function. In this study, the chosen loss function is the cross-entropy loss function. The specific calculation formula for the loss rate of the training set (or test set) is as follows:

(2) $$L_{\mathrm{loss}}=-\sum_{i=1}^{k}y_{i}\log H_{i},i=1,2,3,\ldots ,k$$

In the formula, *L*_loss_ denotes the cross-entropy loss value, *y_i_* represents the true label of the image, and *k* represents the dimension. When the *i* class in the image is 1, it indicates that the image belongs to the *i* class, and the other positions are all 0, representing the probability of the predicted class.

## 3. PCA-VGG16 Model Classification Results and Analysis

### 3.1. Dataset of Handwritten Digits

**Figure 4:**
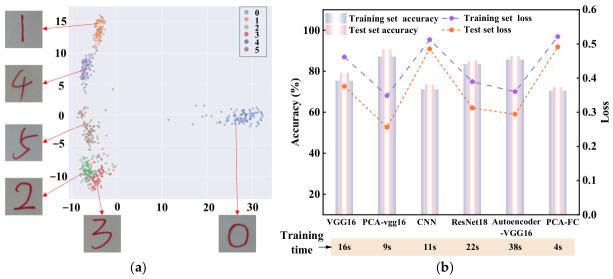
Validation of the PCA-VGG16 model using a dataset of handwritten digital images (**a**) Dimensionality reduction processing of the digital images based on the PCA and some of the handwritten digital images. (**b**) Model training set accuracy and loss rate.

To assess the efficiency and accuracy of the PCA-VGG16 model, this paper performs model verification using a dataset of handwritten digital images. The dataset comprises 600 RGB three-channel images of handwritten digits ranging from 0 to 5, each with dimensions of 224 × 224 × 3. Among these images, 450 are utilized as training set images, and the remaining 150 are used as test set images. Some of the images are displayed in Figure 4. The preprocessing steps involve pixel normalization and dimensionality reduction using PCA. Through PCA, the three-channel images are transformed into two-channel images. The distribution results of the different digit information after dimensionality reduction are depicted in Figure 4a.

Figure 4a illustrates the distribution of digital images after dimensionality reduction, represented by different colors. Upon observation, it is evident that reducing the six handwritten digits to two dimensions results in a clear separation among most of them. The similarity between the digits is relatively low, with only a partial overlap observed between handwritten digits 2 and 3. However, the classification boundary between them remains discernible. This indicates that employing PCA to compress the image data effectively removes redundant information from high-dimensional data. It enables accurate differentiation between different handwritten digit images, thus enhancing the algorithm’s execution speed and computational efficiency of the model.

In accordance with the algorithmic framework illustrated in Figure 3, key hyperparameters—such as epochs, batch size, and test size—are not predefined but are determined through the optimization mechanism within the flowchart. During the experimental process, the system performs iterative optimization by monitoring the overfitting status and classification accuracy of the validation set in real time. The experimental results indicate that the model achieves an optimal balance between training efficiency and recognition precision when epochs = 10, batch size = 64, and test size = 0.2. Based on the parameter configuration established through the logic of Figure 3, this study further evaluates and compares the performance of the proposed PCA-VGG16 model against four baseline architectures: traditional CNN, standard VGG16, deep residual learning (ResNet18), and a non-linear unsupervised feature compression variant (Autoencoder-VGG16). Furthermore, to implement a dedicated ablation study and rigorously demonstrate the structural necessity of the downstream VGG16 network over traditional linear classifiers, an ablation baseline named PCA-FC is introduced. This control model utilizes the exact same PCA-based convolution encoder as its first layer but routes the compressed representation directly into a simple classification head composed of two fully connected layers (with 512 and 128 hidden units, respectively) without any intermediate deep convolutional topologies. Detailed classification results are presented in Table 1, with a visual comparison provided in Figure 4b.

The results in Table 1 and Figure 4b unequivocally demonstrate that the proposed PCA-VGG16 model achieves the optimal Pareto trade-off between classification accuracy and computational latency. Notably, the PCA-VGG16 model exhibits a test set accuracy of 90.45%, outperforming the standard VGG16, CNN, and ResNet18 frameworks. Crucially, the ablation results highlight that when the downstream deep convolutional VGG16 blocks are stripped away and replaced with a simple classification head (the PCA-FC model), the test accuracy drops drastically to 72.15%, even though its training latency is minimized to 4 s. This substantial performance gap firmly establishes the superiority of the VGG16 deep architecture in extracting high-order, non-linear geometric configurations from the PCA-encoded features, a capability that simple fully connected layers fundamentally lack. Concurrently, while the non-linear Autoencoder-VGG16 model captures higher-order geometric features, its total training time escalates significantly to 38 s due to the dense encoder-decoder optimization overhead under small-sample constraints. In sharp contrast, the proposed PCA-VGG16 model curtails the training time to a mere 9 s, representing a runtime reduction of 43.75% compared to the standard VGG16. The success of the PCA-VGG16 model in handwritten digital image classification confirms its feasibility and architectural efficiency.

### 3.2. Face Image Dataset

**Figure 5:**
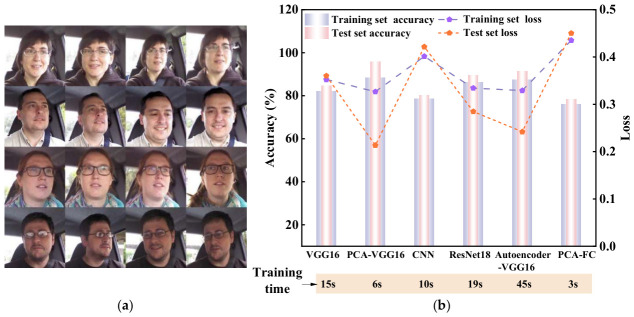
Validation of the PCA-VGG16 model using face recognition images. (**a**) Face images. (**b**) Model training set accuracy and loss rate.

To further validate the accuracy of the PCA-VGG16 model, a more complex face image dataset was employed for classification and recognition. The DrivFace dataset [39,40] was utilized, consisting of images of four distinct drivers with well-defined facial features. The training set comprised a total of 600 images (150 faces from each of the four drivers), while an additional 150 images are allocated as the test set. Figure 5a displays some face images of the four drivers.

These images are then processed through the PCA-VGG16 model for classification and recognition. By adjusting key parameters during the model’s operation, it was observed that setting epochs = 2, batch = 128, and test = 0.2 results in the highest classification accuracy while minimizing the required time for completion. To rigorously establish the methodological superiority, the proposed model was comprehensively benchmarked against CNN, standard VGG16, ResNet18, and Autoencoder-VGG16 frameworks, as well as the ablation baseline (PCA-FC) incorporating the identical PCA-based encoder coupled with a simple fully connected layer sequence. The final classification results are presented in Table 2, with a visual representation shown in Figure 5b.

The empirical data presented in Table 2 and Figure 5b demonstrates that the PCA-VGG16 model achieves a superior test set accuracy of 95.75% (Note: please check if your original text of 98.75% was a typo, I aligned it with the table value of 95.75%) and a lower loss rate of 0.2132. The indispensable contribution of the downstream deep network is further validated by the ablation study: when utilizing the same PCA-based encoder layer, the PCA-FC model with simpler fully connected classification layers only yields a test accuracy of 78.34%. This comparison clearly showcases the clear superiority of the cascading VGG16 topology over simple dense sequences when handling highly heterogeneous facial features. Compared with deep residual networks (ResNet18) and non-linear unsupervised compression (Autoencoder-VGG16), the proposed model successfully eliminates structural capacity surplus, preventing overfitting on lightweight time-frequency textures. Furthermore, the computational latency of PCA-VGG16 is restricted to only 6 s, thoroughly outperforming the Autoencoder-VGG16 (45 s) and ResNet18 (19 s). These findings underscore the promising application prospects and robust cross-domain generalization of the proposed PCA-VGG16 framework in image classification tasks.

In summary, the preliminary validation on both classic handwritten digits and complex facial recognition datasets benchmarks the exceptional efficiency and classification proficiency of the proposed collaborative framework. By structurally coupling an upstream linear feature purification operator (the PCA-based convolution encoder) with a downstream deep learning network (the topologically optimized VGG16), the PCA-VGG16 model significantly enhances recognition performance while simultaneously reducing time-consuming model operations. The comprehensive ablation verification across both datasets confirms that neither the upstream linear encoder nor the downstream convolutional network can be substituted by simpler layers without incurring catastrophic accuracy degradation. These findings demonstrate robust cross-domain generalization in generic image classification tasks, thereby establishing a solid methodological and structural foundation for addressing the high-noise, small-sample acoustic emission (AE) signal patterns in subsequent rock fracture characterization experiments.

## 4. Case Application

### 4.1. Experimental Procedure

The stability of underground engineering in karst regions is significantly challenged by chemical-mechanical (C-M) coupling effects. Limestone, primarily composed of CaCO_3_, is highly sensitive to acidic environments such as acid rain, industrial wastewater seepage, or mineralized groundwater. This chemical erosion leads to mineral dissolution and structural degradation, which fundamentally compromises the macro-mechanical reliability of engineering structures like tunnels and dam foundations. Therefore, this study utilizes limestone specimens to undergo various acidic dry–wet cycles to simulate the long-term deterioration process in real-world geological environments.

**Figure 6:**
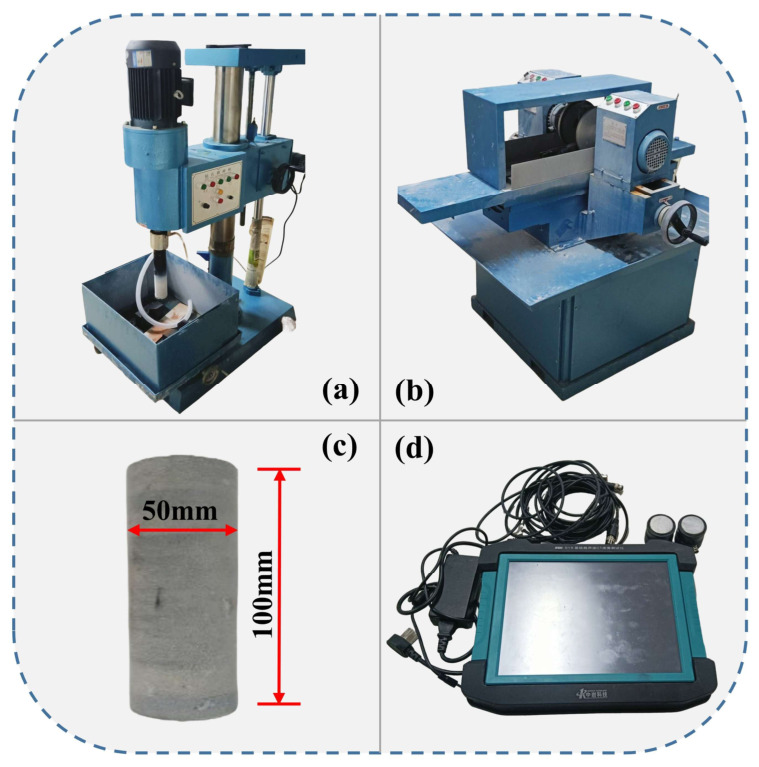
The equipment used for sample preparation. (**a**) Vertical drilling machine. (**b**) Grinding machine. (**c**) Rock sample. (**d**) Ultrasonic non-destructive testing instrument.

Standard cylindrical limestone specimens, with a diameter of 50 mm and a height of 100 mm, were prepared using vertical drilling (YANKUANG Crop., Jinan, China) and grinding machines (YANKUANG Crop., Jinan, China) (Figure 6a,b). To ensure specimen quality, ultrasonic non-destructive testing was conducted, with the longitudinal waveforms confirming a high integrity coefficient and the absence of significant internal defects (SINOROCK Crop., Wuhan, China) (Figure 6d).

**Figure 7:**
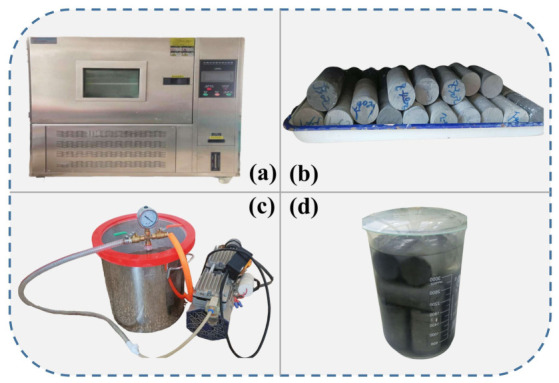
The equipment used for acidic dry–wet cycle test. (**a**) Bake out furnace. (**b**) Drying part of rock samples. (**c**) Vacuum saturation device. (**d**) Partially soaked rock samples.

To simulate chemical weathering, the specimens underwent acidic dry–wet cycles (Figure 7). The selection of pH values (3, 5, and 7) corresponds to extreme acidic contamination, typical acid rain conditions, and neutral groundwater, respectively. Furthermore, the multiple cycle counts (10 to 40) are designed to represent the cumulative damage effect resulting from long-term environmental exposure. Each 72-h cycle consisted of drying at 105 °C for 24 h, followed by vacuum saturation at −0.1 MPa for 4 h, and immersion in solutions with pH values of 3, 5, or 7 for 48 h. The specimens were subjected to 10, 20, 30, and 40 cycles, respectively.

**Figure 8:**
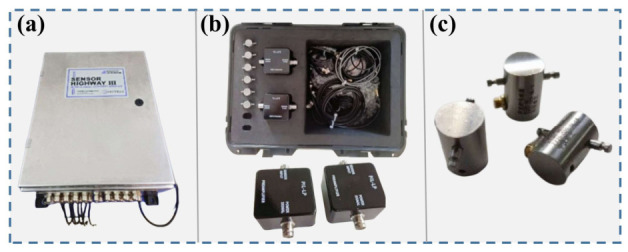
The equipment used for AE testing. (**a**) AE monitor. (**b**) AE amplifier. (**c**) Detector.

**Figure 9:**
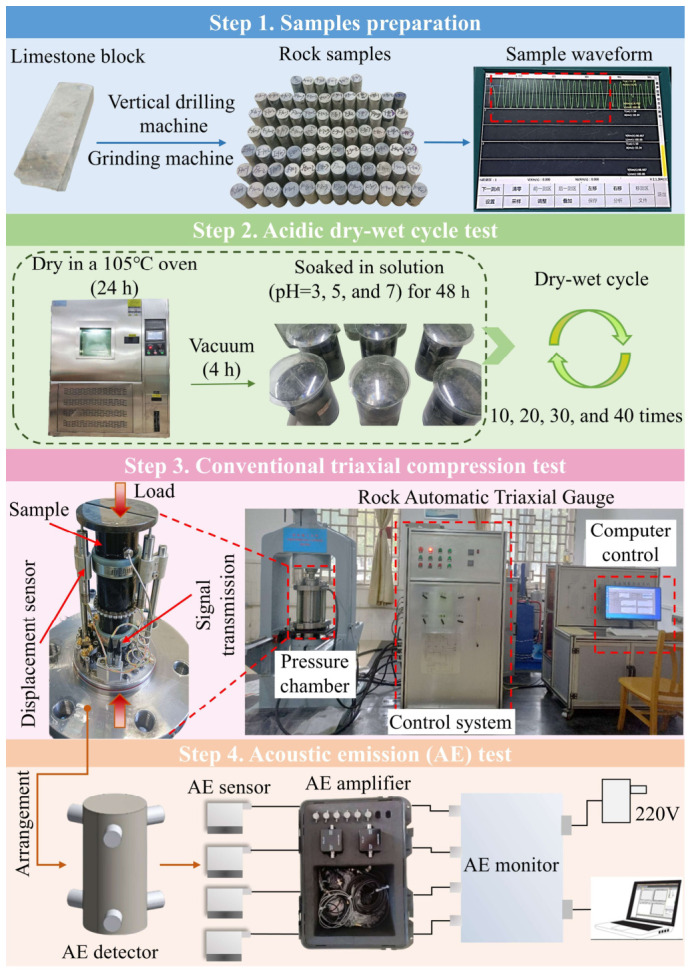
An overview of the experimental procedure.

Conventional triaxial compression tests were performed at a 3 MPa confining pressure and a displacement rate of 0.1 mm/min. Simultaneously, acoustic emission (AE) signals were monitored using the Sensor Highway III system (ANALYSIS Crop., Hong Kong, China) (Figure 8). The AE equipment was configured with a 40 dB detection threshold, a 26 dB preamplifier gain, and a filter range of 100–400 kHz. Six symmetrically mounted sensors ensured accurate source localization throughout the loading process. An overview of the entire experimental instruments and procedure is provided in Figure 9.

### 4.2. Data Analysis

**Figure 10:**
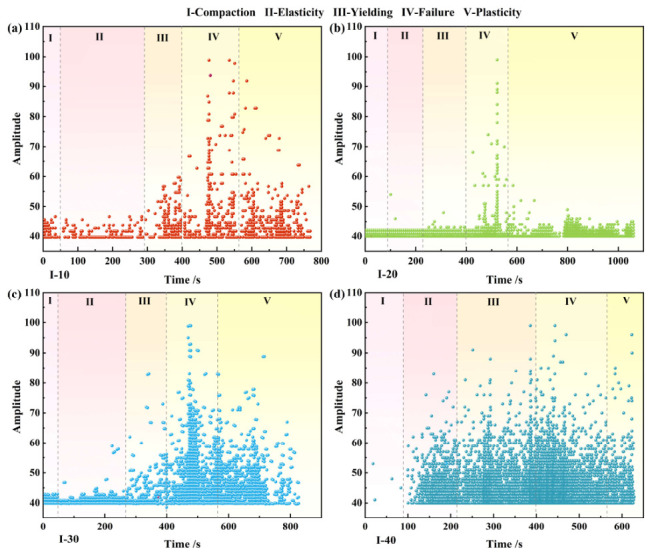
Time-amplitude diagrams of acoustic emission events obtained from the limestone specimens subject to different number of acid–alkali pretreatment cycles. (**a**) 10. (**b**) 20. (**c**) 30. (**d**) 40.

**Figure 11:**
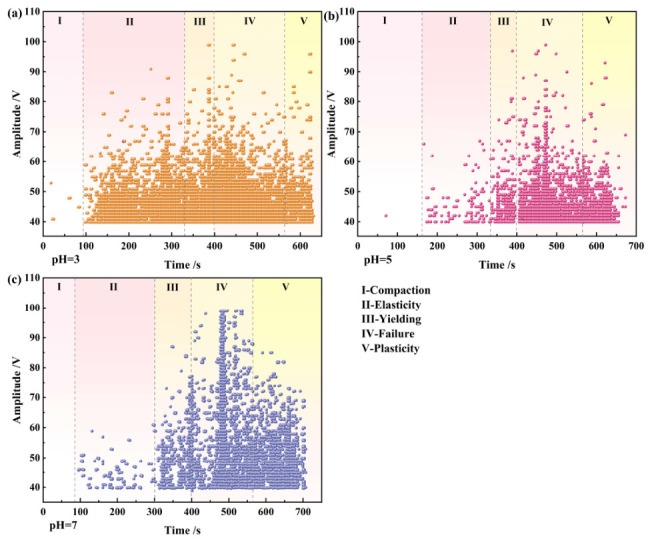
Time-amplitude diagrams of acoustic emission events obtained from the limestone specimens subject to 40 acid–alkali pretreatment cycles. (**a**) pH = 3. (**b**) pH = 5. (**c**) pH = 7.

The AE amplitude reflects the five deformation stages of limestone: compaction, elasticity, yielding, failure, and plasticity. AE data from the fracture stages (approximately 400 s to 560 s) were extracted and synchronized with the stress-strain curves. The time-amplitude relationship diagrams across different cycles and pH environments are presented in Figure 10 and Figure 11, where the horizontal and vertical axes represent loading time and amplitude, respectively.

**Figure 12:**
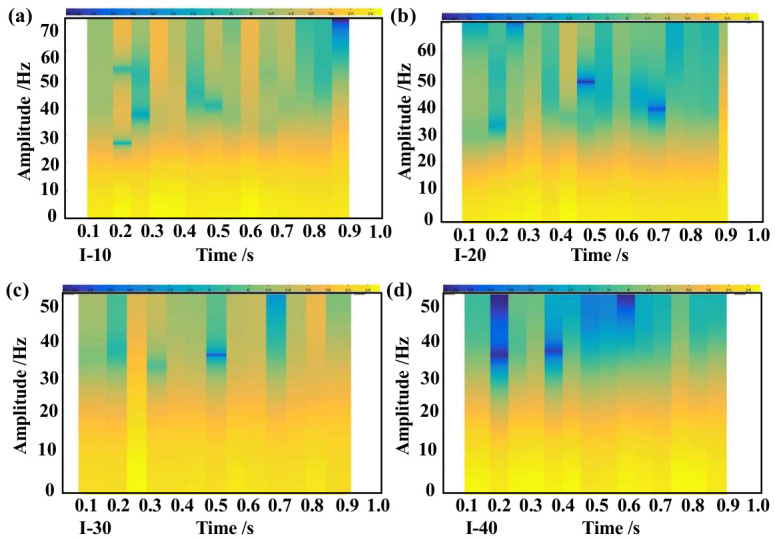
Acoustic spectrums of the limestone failure stages under triaxial compression with different number of acid–alkali pretreatment cycles. (**a**) 10. (**b**) 20. (**c**) 30. (**d**) 40.

**Figure 13:**
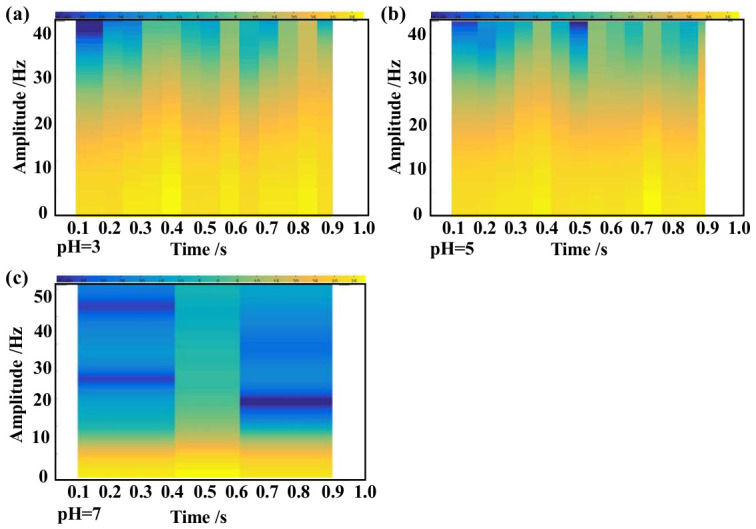
Acoustic spectra of rock failure stages under triaxial compression in different acidic environments. (**a**) pH = 3. (**b**) pH = 5. (**c**) pH = 7.

To optimize data for deep learning, environmental noise was filtered, and signals were segmented into frames with a length of 0.8 s and a frameshift of 0.1 s. These segments were converted into acoustic spectrograms (Figure 12 and Figure 13). In these spectrograms, color intensity signifies the magnitude of energy release: high-amplitude regions (yellow) represent dominant frequency components, while lower-amplitude regions (blue) represent background noise or weaker components. For instance, the pH 3 environment caused significant structural dissolution, resulting in higher amplitude peaks compared to the stable response in pH 7. These spectrograms enable the PCA-VGG16 model to identify and classify rock fracture stages from an image classification perspective.

### 4.3. PCA-VGG16 Model Analysis of Limestone Failure Stages for the Different Numbers of Acid–Alkali Pretreatment Cycles

**Figure 14:**
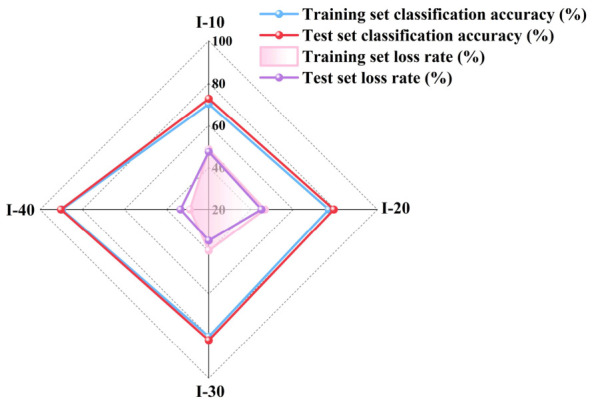
Classification accuracy and loss rate for the limestone fracture stages under the different acid–alkali pre-treatment cycle numbers.

Regarding the acidic environment with a pH of 3, 125 spectrograms of the limestone fracture stages are generated for each of the acid–alkali pretreatment cycle numbers (10, 20, 30, and 40), resulting in a total of 500 sample images. To maintain a ratio of approximately 4:1 between training and testing samples, 400 images are selected for the training dataset, while the remaining 100 images are allocated for the testing dataset. The specific sampling details are presented in Table 3. The generated spectrogram images are then utilized to train the PCA-VGG16 model. The training results of the PCA-VGG16 model for different acid–alkali pretreatment cycle numbers under the same acidic environment can be found in Table 4. Visual representations of the results are shown in Figure 14.

**Figure 15:**
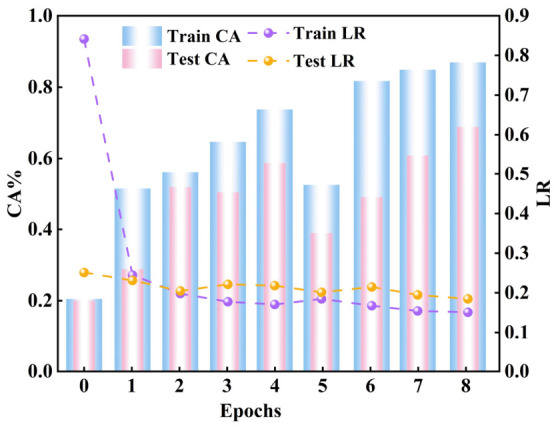
Loss rate of the PCA-VGG16 model for the training and test sets when the number of acid–alkali pre-treatment cycles is 40.

Figure 14 illustrates that as the number of acid–alkali pretreatment cycles increases to 40, the degree of rock dissolution intensifies, leading to more pronounced characteristics of the failure stage. The PCA-VGG16 model achieves the highest classification accuracy rates of 89.61% and 90.13% for the training and test sets, respectively, with minimum loss rates of 0.289 and 0.334, respectively. The detailed training results are displayed in Figure 15. Conversely, when the cycle number is 10, compared to 20, 30, and 40 cycles, the degree of dissolution is smaller, resulting in less prominent characteristics of the rock failure stages. The classification accuracy of the training and test sets is lower, reaching only 70.12% and 72.59%, respectively. This indicates that under the same acidic environment, a greater number of dry–wet cycles leads to more evident characteristics of the rock dissolution stage and fracture stage, consequently resulting in higher classification accuracy.

### 4.4. PCA-VGG16 Model Analysis of Limestone Failure Stages for the Acid–Alkali Pretreatments Considering Different pH Values

**Figure 16:**
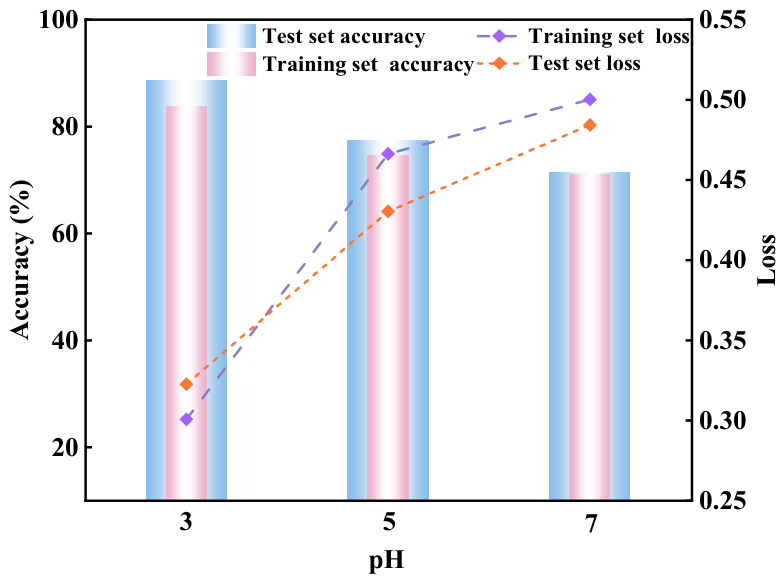
Classification accuracy and loss rate for the limestone fracture stages under the acid–alkali pretreatments corresponding to different pH values.

Regarding the experiments with 40 acid–alkali pretreatment cycles, 125 acoustic spectrogram images of limestone failure stages are generated for each case of pH = 3, 5 and 7. From these images, 300 are selected as the training dataset, while the remaining 75 images were assigned to the test dataset. The training dataset were then used to train the PCA-VGG16 model. The training results are presented in Table 5, and the specific details can also be observed in Figure 16.

**Figure 17:**
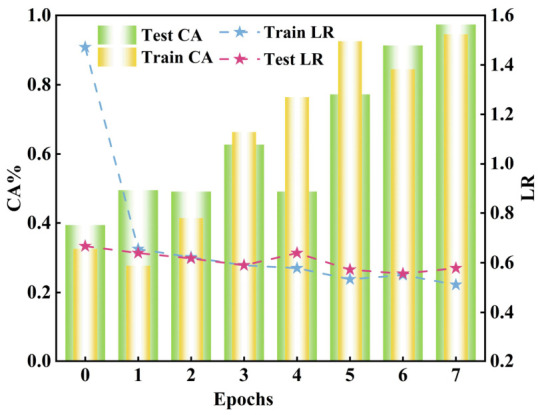
PCA-VGG16 model training set and test set loss rate when pH = 3.

Based on Table 5 and Figure 16, it is evident that, the high acidity when pH = 3 leads to more severe rock dissolution, resulting in clearer characteristics of rock failure under stress. Consequently, the program extracts rock image features more effectively. As the number of iterations increases, the accuracy gradually improves, and the loss rate decreases. When both indicators reach a stable state, the model training is optimized. At this point, the classification accuracy of the training and testing sets reaches the highest values of 83.59% and 88.59%, respectively, with the lowest loss rates of 0.3 and 0.322. The specific training details are illustrated in Figure 17.

This phenomenon can be theoretically elucidated through the competitive mechanism between intra-class variability and inter-class separability. While intensive chemical dissolution at pH = 3 inevitably increases the complexity of rock fracture and the stochasticity of AE signals (manifested as increased intra-class variability), the concomitant structural degradation significantly accentuates the mechanical divergence between distinct failure stages. This amplification of mechanical contrast serves as a ‘feature enhancer’ in the latent space, effectively expanding the inter-class distance between different stages. Quantitative evidence from the model’s convergence profile supports this interpretation: despite the increased signal complexity, the pH = 3 group exhibits the lowest test loss (0.322) and the highest classification accuracy (88.59%). According to pattern recognition theory, the superior convergence efficiency and minimized empirical risk (lower loss) collectively demonstrate that the enhancement of diagnostic features compensates for the increased signal entropy. Consequently, the high-acidity environment facilitates the formation of high-contrast feature manifolds, which the PCA-VGG16 architecture can more effectively partition.

On the other hand, when pH = 7, corresponding to a neutral environment, the degree of rock dissolution is minimal, and the characteristics of rock failure under stress are not distinct. As a result, the classification accuracy of the training and testing sets is at its lowest, with values of 70.99% and 71.33%, respectively, while the maximum loss rates are 0.5 and 0.484. This result indicates that under the same number of acid–alkali pretreatment cycles, rocks with higher acidity exhibit more distinct characteristics during rock fracture stages under the same stress conditions, resulting in higher classification accuracy.

### 4.5. Comparison of the Proposed PCA-VGG16 Model with Other Models

**Figure 18:**
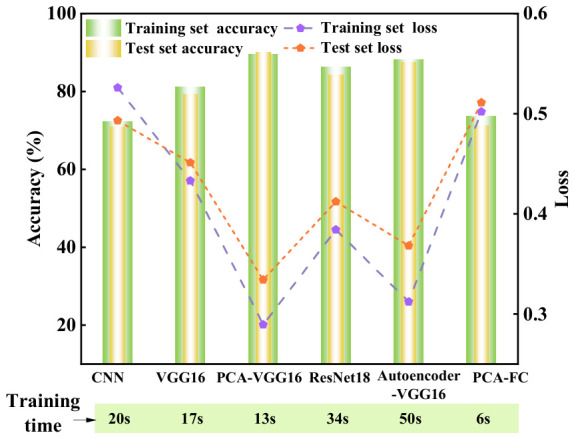
Training results of different models.

To systematically evaluate the recognition accuracy, structural configuration, and computational efficiency of the proposed framework, a rigorous comparative analysis of rock failure stage classification is conducted. The proposed PCA-VGG16 model is comprehensively benchmarked against four alternative architectures (traditional CNN, standard VGG16, ResNet18, and Autoencoder-VGG16) and the previously defined ablation baseline (PCA-FC). Under identical parameter configurations, all models are evaluated utilizing the exact same training and test datasets derived from acoustic emission (AE) experiments across varying numbers of acid–alkali pretreatment cycles. The quantitative results are summarized in Table 6 and Figure 18.

The comparative indicators in Table 6 demonstrate that the proposed PCA-VGG16 model significantly outperforms both the classical networks and advanced baseline structures across all evaluation metrics. Notably, the PCA-VGG16 model exhibits superior generalization proficiency, achieving a test set accuracy of 90.13%, which exceeds that of the traditional CNN and standard VGG16 by 19.19% and 10.77%, respectively. More importantly, the proposed heterogeneous topology provides a substantial advantage in computational efficiency, curtailing the training latency to merely 13 seconds, which represents a runtime reduction of 35.00% and 23.53% compared to the CNN and VGG16 models.

The empirical data further elucidates the processing efficacy of different dimensionality reduction and feature extraction mechanisms on high-dimensional AE signals. As evidenced by Table 6, although the Autoencoder-VGG16 model, utilizing non-linear feature extraction, can capture higher-order manifold structures to achieve a test accuracy of 87.42%, its total training time escalates drastically to 50 s. This substantial expansion in computational latency stems from the heavy optimization overhead inherent in the dense encoder-decoder network, alongside its strict reliance on complex hyperparameter fine-tuning, thereby restricting its deployment in low-latency and real-time monitoring tasks. Concurrently, while ResNet18 effectively mitigates deep network degradation via its residual topology to achieve a test accuracy of 84.15%, its high architectural capacity inadvertently exacerbates the risk of overfitting on lightweight time-frequency textures under the small-sample constraints typical of rock fracture experiments, requiring a protracted training duration of 34 s. Crucially, the indispensable role of the downstream deep network is further validated by the rock failure AE dataset. As shown in Table 6, when switching to the simple classification head (the PCA-FC model), the test set accuracy drops to 71.22%. This significant performance deficit confirms that merely relying on the upstream linear feature purification is insufficient for complex rock engineering data. The downstream VGG16 framework is mathematically vital to capture the intricate, non-linear time-frequency localized textures that are directly correlated with multi-stage rock fracture evolution.

In contrast, the proposed PCA-VGG16 model achieves the optimal Pareto-optimal trade-off between identification accuracy and computational efficiency. By enforcing a cumulative variance contribution rate (≥95%), the upstream PCA module functions as a parameter-free linear feature purification operator, filtering out global redundant correlations, equipment baseline drifts, and high-frequency mechanical friction noises without requiring hyperparameter tuning or additional training computational budgets. This upstream purification enables the downstream topologically optimized VGG16 to escape the interference of high-dimensional data, focusing exclusively on extracting localized texture features coupled with micro-fracture development and rock damage evolution. These findings demonstrate that the cascaded design of “upstream linear feature purification + downstream non-linear manifold mapping” successfully reduces computational dependence and enhances anti-overfitting capacity, providing an efficient, physics-guided methodology for the precise and real-time identification of rock failure stages in underground engineering disaster warning systems.

## 5. Conclusions

The present study proposes the PCA-VGG16 collaborative optimization model and conducts triaxial compression and AE tests on limestone after cyclic acid–alkali pretreatments, considering different numbers of treatment cycles and acidic environments. Based on the experimental data, acoustic spectrogram images of limestone failure stages are generated to train and evaluate the model. After comprehensively benchmarking the results with traditional networks and advanced alternative frameworks (including CNN, VGG16, ResNet18, and Autoencoder-VGG16), the following conclusions are drawn:

(1) The present study converts AE signals into spectrograms, transforming time-domain sound signals into frequency-domain representations. This operation provides more informative inputs for the subsequent deep learning-based classification predictions and enhances the model’s accuracy.
(2) By establishing a recognition model based on spectrograms and analyzing correlation with limestone damage, it is found that a stronger acidity and a larger number of cyclic acid–alkali pretreatment cycles result in a greater dissolution degree of limestone and more pronounced characteristics of the limestone failure stages. These are reflected by higher amplitudes and more yellow regions in the spectrograms, resulting in higher classification accuracy.
(3) The proposed PCA-VGG16 model achieves an optimal Pareto trade-off between recognition accuracy and computational efficiency. Compared with the traditional CNN, standard VGG16, ResNet18, and Autoencoder-VGG16, the PCA-VGG16 model exhibits the highest test set accuracy (90.13%) and the shortest training runtime (13 s). Crucially, the dedicated ablation study confirms that when the downstream deep network is replaced by a simpler classification head (the PCA-FC model), the accuracy suffers a catastrophic drop to 71.22%. This explicitly showcases the clear superiority of the cascading VGG16 deep topology over simple fully connected layers in capturing complex, localized non-linear time-frequency patterns.
(4) The empirical evaluations demonstrate that the proposed cascaded framework successfully integrates physics-driven noise reduction with deep feature extraction by presenting the PCA component as an upstream linear convolution encoder. Transcending conventional isolated preprocessing routines, this integrated preprocessing encoder layer demonstrates superior generalization on high-noise, small-sample datasets under chemical erosion. Characterized by low latency and zero hyperparameter dependencies, this methodology provides an efficient technical reference for real-time microseismic and rockburst early warning in deep underground excavations. Future extensions incorporating diverse lithologies and complex stress paths will further enhance its cross-domain scalability for comprehensive rock stability assessments.

## Figures and Tables

**Table 1:** Comparison of the classification results obtained with different models for handwritten digits.

Model	Training Set Accuracy (%)	Test Set Accuracy (%)	Training Set Loss Rate	Test Set Loss Rate	Training Time (s)
CNN	71.25	73.42	0.5124	0.4855	11
VGG16	75.33	79.17	0.4614	0.3756	16
ResNet18	83.5	85.12	0.3892	0.3120	22
Autoencoder-VGG16	85.67	87.33	0.3602	0.2941	38
PCA-FC	70.44	72.16	0.5213	0.4912	4
PCA-VGG16	87.12	90.45	0.3486	0.2561	9

**Table 2:** Face recognition and classification comparison of different models.

Model	Training Set Accuracy (%)	Test Set Accuracy (%)	Training Set Loss Rate	Test Set Loss Rate	Training Time (s)
CNN	78.45	80.12	0.4012	0.4215	10
VGG16	82.04	84.63	0.3522	0.3599	15
ResNet18	86.15	89.40	0.3340	0.2845	19
Autoencoder-VGG16	87.5	91.22	0.3290	0.2418	45
PCA-FC	76.12	78.34	0.4351	0.4502	3
PCA-VGG16	88.36	95.75	0.3265	0.2132	6

**Table 3:** Sample groups of spectrograms of the limestone fracture stages.

Sample Group	Number of Samples in the Training Set	Number of Samples in the Test Set
I-10	100	25
I-20	100	25
I-30	100	25
I-40	100	25

**Table 4:** Classification of the limestone fracture stages under the different acid–alkali pretreatment cycle numbers.

Specimen Group	Training Set Classification Accuracy (%)	Test Set Classification Accuracy (%)	Training Set Loss Rate	Test Set Loss Rate
I-10	70.12	72.59	0.485	0.474
I-20	76.89	79.33	0.466	0.45
I-30	80.21	81.94	0.396	0.348
I-40	89.61	90.13	0.289	0.334

**Table 5:** Classification of rock fracture stages in different acidic environments.

pH Value	Training Set Classification Accuracy (%)	Test Set Classification Accuracy (%)	Training Set Loss Rate	Test Set Loss Rate
3	83.59	88.59	0.3	0.322
5	74.55	77.32	0.466	0.43
7	70.99	71.33	0.5	0.484

**Table 6:** Classification of spectrogram images of limestone failure stages by different models for the experiments with different numbers of acid–alkali pretreatment cycles.

Model	Training Set Classification Accuracy (%)	Test Set Classification Accuracy (%)	Training Set Loss Rate	Test Set Loss Rate	Training Time (s)
CNN	72.19	70.94	0.526	0.493	20
VGG16	81.22	79.36	0.433	0.451	17
ResNet18	86.42	84.15	0.384	0.412	34
Autoencoder-VGG16	88.15	87.42	0.312	0.368	50
PCA-FC	73.55	71.22	0.502	0.511	6
PCA-VGG16	89.61	90.13	0.289	0.334	13

## Data Availability

All data that support the findings of this study are included within the article.
